# Compliance with Dietary Guidelines Varies by Weight Status: A Cross-Sectional Study of Australian Adults

**DOI:** 10.3390/nu10020197

**Published:** 2018-02-11

**Authors:** Gilly A. Hendrie, Rebecca K. Golley, Manny Noakes

**Affiliations:** 1 CSIRO Health and Biosecurity, P.O. Box 10041, Adelaide, SA 5000, Australia; manny.noakes@csiro.au; 2 School of Pharmacy and Medical Sciences, University of South Australia, Adelaide, SA 5000, Australia; rebecca.golley@flinders.edu.au

**Keywords:** diet quality, dietary guidelines, obesity, discretionary choices

## Abstract

Population surveys have rarely identified dietary patterns associated with excess energy intake in relation to risk of obesity. This study uses self-reported food intake data from the validated Commonwealth Scientific and Industrial Research Organisation (CSIRO) Healthy Diet Score survey to examine whether apparent compliance with dietary guidelines varies by weight status. The sample of 185,951 Australian adults were majority female (71.8%), with 30.2%, 35.3% and 31.0% aged between 18–30, 31–50 and 51–70 years respectively. Using multinomial regression, in the adjusted model controlling for gender and age, individuals in the lowest quintile of diet quality were almost three times more likely to be obese than those in the highest quintile (OR 2.99, CI: 2.88:3.11; *p* < 0.001). The differential components of diet quality between normal and obese adults were fruit (difference in compliance score 12.9 points out of a possible 100, CI: 12.3:13.5; *p* < 0.001), discretionary foods (8.7 points, CI: 8.1:9.2; *p* < 0.001), and healthy fats (7.7 points, CI: 7.2:8.1; *p* < 0.001). Discretionary foods was the lowest scoring component across all gender and weight status groups, and are an important intervention target to improve diet quality. This study contributes to the evidence that diet quality is associated with health outcomes, including weight status, and will be useful in framing recommendations for obesity prevention and management.

## 1. Introduction

Obesity is a major risk factor for non-communicable disease including cardiovascular disease, diabetes and some cancers [1]. Obesity is at pandemic levels, affecting 10–15% of the global population [2], and up to one-third of adults in developed countries such as Australia and the United States of America (USA) [3,4]. Diet, specifically excess energy intake relative to energy expenditure, is a key modifiable cause of obesity [5]. 

It has been a challenge to identify the dietary patterns clearly linked to excess energy intake [6]. Research on dietary patterns aims to capture the behavioural complexity of food and beverage intake combinations that underpin the associations between diet and health [7,8]. While significant associations between dietary patterns and weight status are observed, these findings are weak and inconsistent and warrant further investigation [6,8,9].

Dietary indexes are a composite indicator of diet quality, where adherence to an *a priori* set of components or recommendations is reflected in a single score [8]. Indexes can incorporate dietary quality, diversity, adequacy, moderation and balance [10,11]. Some indexes are nutrient-based, others food-based, or a combination of both [12]. In the context of obesity, diet quality could be defined as the degree to which a dietary pattern reduces the risk of positive energy balance. In developed countries, diet quality tools measuring adherence to national dietary guidelines are consistently inversely associated with obesity [9]. For example in the USA, the Healthy Eating Index is inversely associated with weight status in 10 of 13 studies examined in a recent systematic review [9]. Similarly in Australian research, dietary guideline adherence measured using the Dietary Guideline Index (DGI) has shown similar associations [13,14]. Conversely, diet quality conceptualised as dietary diversity has been positively associated with risk of obesity [9]. A priori components selected for inclusion in a diet quality score is likely to influence the utility of diet quality measures in obesity research.

In 2016, Livingstone and McNaughton compared the association between two food-based diet quality scores, the DGI and the Recommended Food Score (RFS), and obesity [15]. The DGI includes components reflecting adherence to recommendations for core food groups—‘healthy foods’—and discretionary choices—‘unhealthy food and beverages’. In contrast, the RFS conceptualises diet quality as variety of ‘healthy’ or core foods only. When the DGI and RFS scores were applied to nationally representative food intake data of Australian adults, only the DGI score was associated with lower risk of overall and central obesity [15]. The authors concluded that inclusion of both healthy and unhealthy components appears to be important in conceptualising diet quality as a risk factor for obesity. Whether similar findings can be observed for other components of diet quality, for example within the core food groups, remains unexplored. Given the multifaceted nature of diet quality indexes, there remains unanswered questions around whether particular aspects of diet quality or compliance with guidelines differs between weight status groups. This warrants further investigation.

The DGI reflects compliance with the 2013 Australian Dietary Guidelines and has been applied to food intake data measured via 24 h recalls [15], food frequency questionnaires [16] and a short food survey [17,18]. Regardless of dietary assessment method, the DGI is significantly associated with weight status in large population surveys [15,16,17]. The Commonwealth Scientific and Industrial Research Organisation (CSIRO) Healthy Diet Score survey is a freely available online survey designed to assess diet quality using the DGI scoring approach. The survey allows individuals to enter their food intake and receive immediate feedback in the form of a numerical diet quality score as well as three brief statements on how to improve their score [17]. The CSIRO Healthy Diet Score survey enables the examination of population diet quality in the context of the provision of individualised feedback to improve diet quality. In this paper, we use data from this online survey to explore whether compliance with particular dietary guidelines varies by weight status, and to identify key food groups to target to improve diet quality for different weight status groups.

## 2. Materials and Methods

This paper describes cross-sectional analysis of self-reported food intake data collected using an online short food survey designed to assess diet quality defined as compliance with the Australian Dietary Guidelines. The paper examines differences in compliance with guidelines by weight status in a large sample of Australian adults.

### 2.1. CSIRO Healthy Diet Score Survey

The development and validation of the short food questions and scoring criteria to assess compliance with the Australian Dietary Guidelines [19], as well as the development of the online survey, known as the CSIRO Healthy Diet Score survey [17], have been described in detail previously. Briefly, the CSIRO Healthy Diet Score survey is an extension of the Short Food Survey specifically designed to calculate a Dietary Guideline Index score [13,20] assessing compliance with dietary guidelines (referred to here as “Diet Score”). The ability of the Short Food Survey to assess overall diet quality has undergone validation in a sample of Australian adults [18] and children [21]. The survey is a series of 38 short questions asking individuals to report their usual intake of core (fruit, vegetables, grains, meat and alternatives, and dairy and alternatives) and discretionary (e.g., cakes and biscuits, chocolate and confectionary, takeaway foods, savoury pies and pastries, sugar sweetened beverages, and alcohol) foods and beverages. Individuals report their frequency of consumption as daily, weekly, or monthly, and portion consumed in multiples of standard serving sizes (serves) [19]. The survey also contains questions about the frequency of wholegrains, low fat dairy consumption, fat type of spreads used and trimming of meat, water consumption and variety of core foods consumed. The scoring algorithm compares the daily amount of core and discretionary foods consumed to age and gender specific recommendations in the Australian Dietary Guidelines [19]. A compliance score for each component (scored out of 10 except for discretionary foods which is out of 20) is summed to give an overall Diet Score out of 100. Higher scores reflect better diet quality, conceptualised as greater compliance with the Australian Dietary Guidelines. The survey also includes questions covering demographic characteristics of gender, age, Australian state of residence, and occupation; as well as self-reported height and weight. Height and weight were used to calculate Body Mass Index (BMI) and the World Health Organization International Classifications for adults used to classify weight status. This study was approved by the CSIRO Health and Medical Human Research Ethics Committee Low Risk Review Panel (LR 29/2016).

### 2.2. Data Collection and Preparation

The CSIRO Healthy Diet Score survey was launched on the 21 May 2015 and is freely available online. The survey website is a live website, meaning data collection is continuous and ongoing. The full details of the data collection process have been described [17]. Briefly, five media releases have received national television, print and radio coverage across a range of free to air stations. This paper uses data collected from individuals who completed the survey from the date of launch through to the 15 August 2017. During this time the survey has been commenced 236,276 times. Using a standard process of data cleaning [17], duplicates were removed using an ID variable and taking the first survey attempt from each individual. In addition, partially completed survey and outliers were removed based on extreme age (less than 18 years and greater than 100 years), body mass index (less than 13 and greater than 97), height (less than 1 m and greater than 3 m), and weight (less than 13 kg and greater than 250 kg).

The majority of the sample were female (72.2%), with a relatively even distribution across the 18–30, 31–50, 51–70 year age groups (31.1%, 34.9%, 30.5%). However, the oldest age group of 71+ years were underrepresented relative to the Australian population (3.5% vs. 12.5% [22]). The nature of recruitment resulted in a sample that was under-representative of males and older adults relative to the broader Australian population [22], therefore survey data were weighted by gender and age group. Given the focus on obesity, underweight individuals were not included in this paper (2.6% of the sample), leaving 185,951 individuals for analysis.

### 2.3. Statistical Analysis

Descriptive analysis (means, standard deviations) were used to describe the compliance scores for each component of the Diet Score as well as the Diet Score overall, by gender and weight status. To aid interpretation and comparison of scores between the components, we have expressed the component scores (originally out of 10, except for discretionary foods which was out of 20) as a score out of 100 in the tables and description of results.

Differences in component scores by weight status were calculated as normal weight minus overweight or obese—meaning that positive difference values reflect a higher score or greater compliance with dietary guidelines in normal individuals compared to the overweight or obese individuals. The significance of the differences between weight status groups were explored using One-way Analysis of Variance, with Bonferroni adjustment, and a significance level of *p* < 0.001. Given the large sample size, we also used cut offs of 5% and 10% as a guide to interpret the meaningfulness of the difference between groups. Therefore a statistically significant difference less than five points between weight status groups was considered small, five to less than 10 points was considered a medium difference and ten or more considered a large difference. Discussion of results favoured the most meaningful differences, as opposed to just the statistically significant differences.

To examine the likelihood of obesity by degree of diet quality score, quintiles of Diet Score were created and multinomial logistic regression performed adjusting for age (as a continuous variable) and gender (categorical). The highest quintile of Diet Score was the reference, and the odds ratios represent the risk of overweight and obesity, or just obesity, relative to normal weight. All analysis was conducted in IBM SPSS Statistics 23.

## 3. Results

The majority of the sample (*n* = 185,951) included in this analysis were female (71.8%), and 30.2% were aged between 18–30 years, 35.3% were 31–50 years, 31.0% were 51–70 years and 3.5% aged 71 years or older. After removing underweight individuals, 49.8% of the sample was of a normal weight, 30.6% overweight and 19.7% obese.

### 3.1. Variation in Diet Score by Weight Status

Overall Diet Score decreased from 60.5 in normal weight adults to 58.3 and 55.8 in overweight and obese adults. In males, the Diet Score decreased from 58.4 to 56.5 (a difference of 1.9 points) in normal and overweight adults, and from 58.4 to 53.1 (a difference of 5.4 points) in normal weight and obese adults. In females, Diet Score decreased from 62.1 to 60.7 (difference of 1.8 points) and from 62.1 to 58.0 (difference of 4.5 points) in normal to overweight, and normal to obese adults respectively (Table 1).

### 3.2. Likelihood of Overweight and Obesity by Diet Score Quintile

As Diet Score decreased the likelihood of being obese increased incrementally. In the adjusted model controlling for gender and age, individuals in the lowest quintile of Diet Score were almost three times as likely to be obese than those in the highest quintile (OR 2.99, CI: 2.88:3.11; *p* < 0.001). Similar results were observed for likelihood of overweight or obese (OR 2.64, CI: 2.56:2.73; *p* < 0.001), however the odds ratios were moderated slightly compared to likelihood of obesity (Table 2).

### 3.3. Rank Order of Components of Diet Quality

**Figure 1:**
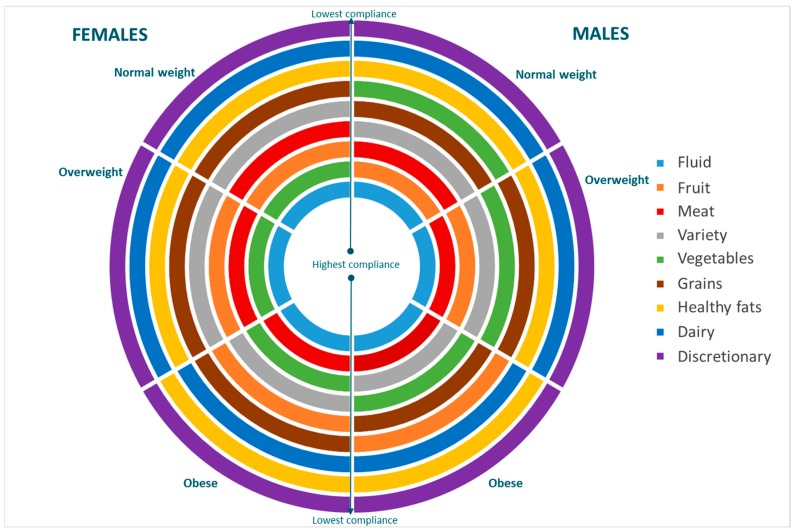
Rank order of Dietary Guideline compliance scores, by gender and weight status.

Discretionary food was the lowest scoring diet quality component across all gender and weight status groups—meaning all Australians were least compliant with the dietary guidelines around discretionary foods and beverages consuming these foods in excess of recommendations (Table 1). While the order differed slightly for obese compared to normal and overweight adults, dairy foods and healthy fats were the next two lowest scoring components (Figure 1).

Fluids was the highest scoring diet quality component across all gender and weight status groups. Regardless of gender, obese adults scored highest for the fluids followed by meat and alternatives, whereas there were some gender differences in the highest scoring components for normal and overweight adults. Normal weight males scored highest for fluids followed by fruit, and overweight males highest for fluid followed by meat. Normal and overweight females both scored highest for fluids, followed by vegetables (Figure 1).

### 3.4. Differences in Diet Quality Compliance Scores by Weight Status and Gender

While there were some similarities in the rank order of the Diet Score components between genders and across weight status groups, there was variation in the absolute values of the component scores (Table 1), and the differences between weight status groups (Table 3). Statistically significant differences in compliance scores between normal weight and overweight, and between normal weight and obese Australians, were observed for all components of diet quality except meat in males, and grains in females. The differences between normal and obese adults were larger than between normal and overweight adults for all food groups except meat, where the component scores were consistent across all weight status groups.

The largest differences were observed between normal weight and obese adults for fruit, with normal weight adults scoring 12.9 points (out of 100) higher than obese adults. This result was similar in males and females. Positive medium differences of between 5 and 10 points (out of 100) were observed for discretionary foods, healthy fats, grains and variety between normal weight and obese males; and for discretionary foods and healthy fats between normal weight and obese females. For healthy fats we also found that normal weight adults scored higher than obese adults on both scoring elements, that is normal weight adults were more likely to always trim their meat and use unsaturated fat containing spreads (data not shown).

**Figure 2:**
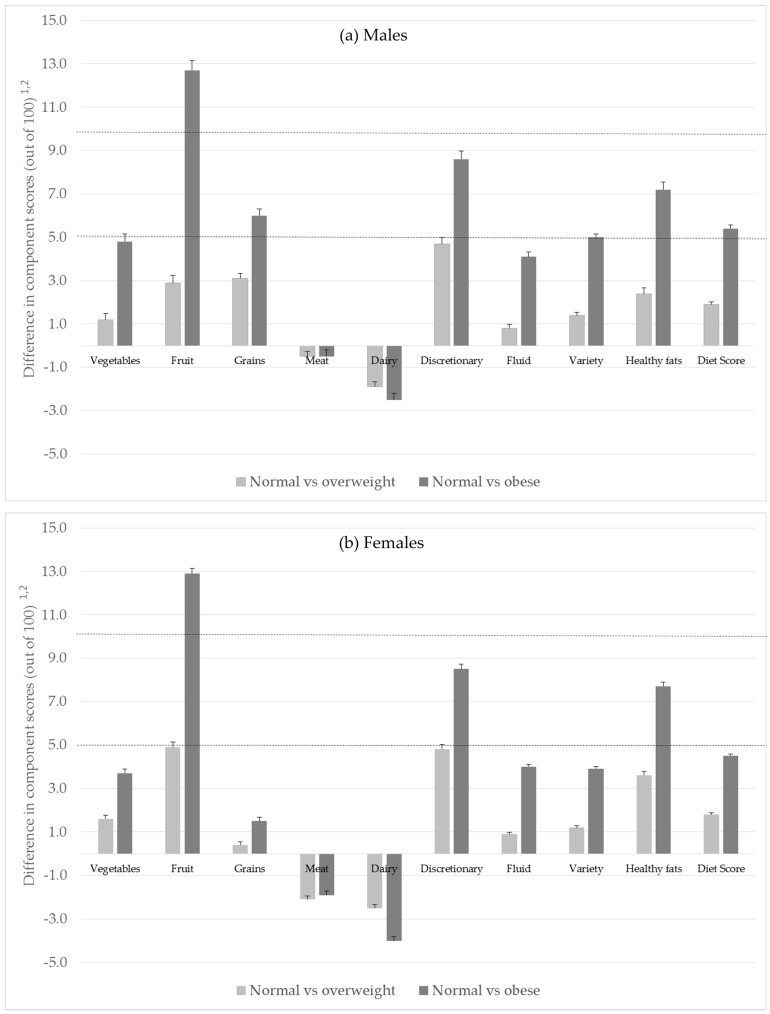
Differences ^1,2^ in compliance scores (mean difference plus standard error) between normal and overweight (light grey), and normal weight and obese adults (dark grey), by gender (**a**) Males, (**b**) Females. ^1^ A positive difference score means normal weight has a higher score and therefore greater compliance with Dietary Guidelines. ^2^ A difference of less than 5 points is considered small, 5 to less than 10 medium, and 10 or more a large difference. The dotted horizontal lines are positioned at 5 and 10 points.

The only negative differences were observed for meat and dairy foods, meaning that overweight and obese adults were more compliant with dietary guidelines than normal weight adults. These were also one of the few food groups where differences between normal weight and obese adults were greater in females than males (Figure 2).

The grains and dairy components also had two scoring elements—one for amount consumed and one for quality. For grains, normal weight adults scored significantly higher on use of wholegrain bread as opposed to white varieties than obese adults (a significant medium difference), but compliance with the servings (or amount) recommendations varied by gender. Normal weight men had a higher average score—meaning they were more compliant with guidelines around the amount of grains to consume than obese men (significant medium effect). Whereas normal weight women were slightly less compliant than obese women (small but significant difference). Obese adults also scored higher on dairy type—meaning they were more likely to report using skim or low fat milk compared to normal weight adults. There was small differences in compliance with the serving recommendation for males, but normal weight and obese females were equally compliant with the serving recommendations for dairy foods (data not shown).

## 4. Discussion

This paper aimed to assess the relationship between the CSIRO Healthy Diet Score and weight status and determine whether it may have utility in framing dietary recommendations for the prevention and management of overweight and obesity. Access to this large dataset has allowed for detailed subgroup analysis, increasing our capacity to understand the specific components of diet quality which differ by individual characteristics, in this case gender and weight status. Given the statistical power of the sample we used cut offs in addition to statistical significance to moderate our interpretation of results, and focus the discussion on results with medium to large effect sizes.

In this large sample of Australian adults who have completed the online CSIRO Healthy Diet Score survey we found that low compliance with dietary guidelines was associated with an almost 3-fold higher likelihood of being obese. We also found that the total score per se may not be as important as the dietary pattern within the score. For example, there was a small difference in overall diet quality score between obese and normal weight individuals, however, moderate to large differences in component scores by weight status category were observed for discretionary foods, fruit and healthy fats. Discretionary foods are an important intervention target to improve diet quality, regardless of weight status or gender.

The relationship between diet quality and weight status has shown mixed results [6,9], and the definition of diet quality appears to be important [9]. In this study, an online diet quality score was used that reflected adherence to the 2013 Australian Dietary Guidelines which was applied to food intake data collection using a short food survey. A key finding was the apparent stepwise increase in likelihood of being classified as overweight or obese with decreasing compliance with dietary guidelines. The findings are consistent with other dietary pattern research that has used the same scoring approach, the Dietary Guideline Index, applied to food intake data derived from a nationally representative sample using 24-h recalls [15] and a large sample of older adults using a Food Frequency Questionnaire [16]. The present study reinforces that diet quality, conceptualized as compliance with guidelines, is a relevant intervention target for obesity prevention and management, and that specific elements of the score could be used to provide personalised feedback to individuals.

It is valuable to understand if specific components of dietary guideline adherence are of particular importance in the context of the overweight and obesity [23]. In this study, components of diet quality that contrasted most by weight status were fruit, discretionary foods, and healthy fats. For men, vegetables, grains and variety component scores also showed moderate differences by weights status groups—all favouring greater compliance with guidelines in normal weight individuals. For women, there were small differences in compliance with the guidelines for dairy but further analysis showed this was due to the fat type of dairy products consumed and not the amount. Interestingly, dairy and meat were the only two food groups for which obese individuals were more compliant with guidelines. While these differences were considered to be small they are worth further investigation. For example, these may reflect true differences in intake but may also reflect the construction of the index. The scoring algorithm does not penalize for overconsumption of healthy foods. Scoring the idea of eating beyond one’s needs, could be explored by applying a bell-shaped scoring system whereby at a threshold of overconsumption scores start to reduce. However, this threshold may need to be food group specific and not follow a general rule, given there is evidence that overconsumption of some food groups, such as meat, may be more detrimental to health [24] than overconsumption of other food groups such as vegetables.

The finding that discretionary foods—also termed unhealthy foods—are an important component of diet quality in the context of obesity are consistent with recent analysis comparing diet quality indexes that are comprised of only healthy food based components compared with those comprised of healthy and unhealthy food based components [15]. The Recommended Food Score is based on consumption of five healthy food groups, and shows no association with obesity risk [15]; likewise dietary diversity indexes are also not associated with obesity [25]. The Australian Dietary Guidelines make population level recommendations for appropriate types and portions of foods to consume for health and wellbeing [19]. In contrast to the US Dietary Guidelines, the Australian guidelines avoid explicitly linking food intake recommendations to set energy requirements. Rather there is a food-based recommendation to limit discretionary foods that are higher in saturated fat, added sugar, alcohol and/or sodium to “sometimes and in small amounts” [19]. This focus on nutrient-poor foods appears to sufficiently address consumption of energy dense foods and prevention of positive energy balance [23].

It is generally accepted that reduction in energy intake is a necessary focus in nutrition interventions targeting obesity. However there is less consensus around specific food group-based strategies required to achieve a moderation in energy intake, and whether these targets are consistent across all subgroups within the population. To improve diet quality, this study suggests that obese individuals need to increase their consumption of fruit, and choose behaviours that improve the quality of grains and dietary fats, that is choosing wholegrains, unsaturated spreads and trimming meat. For men, increasing vegetable consumption and including a wide variety of core foods were also identified as key differentiating factors in diet quality between normal weight and obese adults. Fruit and vegetables initiatives are common targets for obesity prevention and important targets given their low energy density, and association with reduced disease risk [26,27,28]. However, other less explored areas of diet quality such as healthy fats, wholegrains and variety may be additional targets to consider and provide a more nuanced approach to population nutrition and obesity prevention programs. However, in the context of obesity prevention, dietary factors should be considered together with non-dietary factors such as physical activity, as their effects alone on body weight may be small over the longer term. It has proven difficult to separate out the effects of diet from physical activity in weight gain because few nutrition epidemiology studies adequately control for physical activity [29]. Therefore to better understand relationships between dietary components and weight status a broader range of covariates need to be consider such as age and physical activity. Delivering the survey online has resulted in large volumes of data being collected in a time and cost effective way. Exploration of this dataset has identified elements of diet quality associated with increased risk of obesity, which can now be used to inform interventions that address its management and prevention at the population level. The survey platform currently provides brief and immediate feedback to individuals, and the results of this analysis can be used to further refine this feedback to improve the overall diet quality of the population. The opportunity “big data” provides in terms of tailoring feedback is an emerging area of public health research, and moves towards the concept of quantified population health [30]. The online environment also helps to accelerate the progress in intervention development, as it speed up the temporal lag associated with traditional data collection and dissemination cycles [30].

Limitations of our research are that due to the nature of recruitment, being an online food intake survey, males, older adults and obese individuals were underrepresented relative to the Australian population. However, these were partially accounted for in our adjustment for age and gender. While extreme values were removed, self-reported height and weight could have led to an inaccurate weight status classification. The ability to self-report anthropometric data may vary systematically by demographic characteristics such as age, gender and socioeconomic status [31]. Misreporting of food intake can also vary by individual characteristics such as weight status. Underreporting of intake is more frequent and of a greater magnitude in obese individuals [32,33,34], and more likely to be due to underreporting of discretionary foods, which could have led to inaccurate diet quality scores as well.

Another limitation of this study was its cross sectional design, which limits any inference of causal relationships. Therefore longitudinal data are needed to determine if diet quality predicts risk of obesity and/or energy imbalance, and whether changing intake of particular foods groups to be more consistent with guidelines reduces risk of future obesity. In addition, this study did not assess levels of physical activity in relation to diet quality and obesity status. Therefore, future research should also consider energy expenditure, as the other important element of energy balance, as well as other potential confounding factors, including individual and environmental level factors such as socio-economic status, physical activity and accessibility to healthy foods. Regardless, this study adds to the evidence base that diet quality is associated with health outcomes, including weight status.

## 5. Conclusions

The paper uses data from the CSIRO Healthy Diet Score survey to demonstrate differences in dietary patterns by weight status in a large sample of Australian adults. The key discriminating factors of diet quality between normal and obese adults were consumption of fruit, discretionary foods and healthy fats. These insights will allow the provision of more specific advice around dietary targets in subgroups of the population to increase diet quality. Shifting towards a more precision approach to prevention of obesity may be more effective in changing behaviour within the context of population level nutrition recommendations. Utilising technology has enhanced the reach of this dietary assessment tool, and future research will examine whether online self-assessment and tailored dietary feedback is an efficient and cost effective way to improve diet quality.

## Figures and Tables

**Table 1:** Dietary Guideline compliance scores (out of 100) ^1^ by gender and weight status.

	Compliance Score out of 100 (Mean (Standard Deviation))								
	Male (*n* = 52,400)			Female (*n* = 133,551)			Total (*n* = 185,951)		
Food Group	Normal (*n* = 22,101)	Overweight (*n* = 20,761)	Obese (*n* = 9538)	Normal (*n* = 70,410)	Overweight (*n* = 36,073)	Obese (*n* = 27,068)	Normal (*n* = 92,511)	Overweight (*n* = 56,834)	Obese (*n* = 36,606)
Vegetables	64.4 (29.1)	63.2 (29.4)	59.8 (30.7)	76.2 (26.7)	75 (27.5)	72.9 (28.5)	71.2 (28.4)	68.4 (29.2)	67.2 (30.2)
Fruit	70.7 (35.3)	67.7 (36.2)	58.2 (38.8)	74.3 (33)	70.5 (34.8)	62.8 (37.3)	72.8 (34)	69 (35.6)	60.8 (38)
Grains	65.1 (24.7)	62.3 (24.2)	59.2 (25.2)	63.3 (24.5)	63.5 (25.1)	62.6 (25.9)	64.1 (24.6)	62.9 (24.6)	61.1 (25.6)
Meat	68.7 (26.1)	69.0 (25.8)	69.3 (26.1)	72.3 (25.2)	74.4 (24.4)	74.2 (24.4)	70.8 (25.7)	71.4 (25.3)	72 (25.3)
Dairy	46.5 (24.8)	48.3 (25.5)	48.7 (26)	46.7 (26)	49.1 (26.2)	50.5 (26.6)	46.7 (25.5)	48.7 (25.8)	49.7 (26.3)
Discretionary	29.5 (32.1)	24.7 (30.6)	20.9 (29.4)	35.1 (32.7)	30.6 (32)	26.8 (31.6)	32.7 (32.5)	27.3 (31.3)	24.2 (30.8)
Fluid	90.1 (16.6)	89.1 (18)	86.1 (21.5)	94.9 (11.7)	94.2 (13.1)	91.3 (17.2)	92.8 (14.2)	91.4 (16.2)	89.1 (19.4)
Variety	66.3 (13)	64.9 (12.9)	61.4 (14)	67.1 (12.3)	66.1 (12.7)	63.4 (13.6)	66.7 (12.6)	65.5 (12.8)	62.5 (13.8)
Healthy fats	53.1 (29.1)	50.7 (28)	46 (27.8)	56 (26.8)	52.6 (25.6)	48.7 (25.2)	54.7 (27.8)	51.5 (27)	47.5 (26.4)
Diet Score	58.4 (13.1)	56.5 (12.6)	53.1 (13.2)	62.1 (12.2)	60.7 (12.3)	58 (13)	60.5 (12.8)	58.3 (12.7)	55.8 (13.3)

^1^ Compliance score data is weighted by age group and gender to reflect the demographic profile of the general Australian population taken from the 2016 Census data [22].

**Table 2:** Multinomial adjusted odds ratios and 95% confidence internals of overweight and obese per quintile of Diet Score ^1^.

	Diet Score				
Variable	Q1 (Lowest)	Q2	Q3	Q4	Q5 (Highest)
Cases	(*n* = 36,525)	(*n* = 37,134)	(*n* = 37,437)	(*n* = 37,494)	(*n* = 37,068)
Diet score (out of 100)					
Mean	40.3	52.1	59.1	66.0	76.7
Range	3.7–47.8	47.9–55.7	55.8–62.3	62.4–69.9	70.0–99.0
Obese					
Crude odds ratio	2.19 (2.11, 2.27)	1.60 (1.54, 1.66)	1.30 (1.25, 1.35)	1.16 (1.12, 1.21)	1.0
Adjusted model^1^	2.99 (2.88, 3.11)	1.91 (1.84, 1.99)	1.45 (1.39, 1.50)	1.22 (1.17, 1.27)	1.0
Overweight or obese					
Crude odds ratio	2.01 (1.95, 2.07)	1.58 (1.54, 1.63)	1.38 (1.34, 1.42)	1.23 (1.19, 1.26)	1.0
Adjusted model^1^	2.64 (2.56,2.73)	1.85 (1.80, 1.91)	1.52 (1.47, 1.56)	1.28 (1.24, 1.32)	1.0

^1^ Adjusted model is adjusted for gender (categorical variable) and age (continuous variable). All models are significant at *p* < 0.001.

**Table 3:** Difference in Dietary Guideline compliance scores (out of 100) between normal and overweight, and normal and obese adults by gender ^1^.

	Compliance Score Out of 100 (Mean (Standard Deviation))					
	Male (*n* = 52,400)		Female (*n* = 133,551)		Total (*n* = 185,951)	
Food Group	Normal vs. Overweight	Normal vs. Obese	Normal vs. Overweight	Normal vs. Obese	Normal vs. Overweight	Normal vs. Obese
Vegetables	1.2 * (0.5:2.0)	4.8 * (3.8:5.8)	1.6 * (1.1:2.0)	3.7 (3.1:4.2)	2.9 * (2.5:3.3)	4.2 * (3.7:4.7)
Fruit	2.9 * (2.0:3.9)	12.7 * (11.5:13.9)	4.9 * (4.3:5.5)	12.9 (12.2:13.5)	4.6 * (4.1:5.1)	12.9 * (12.3:13.5)
Grains	3.1 * (2.5:3.7)	6.0 * (5.2:6.8)	0.4 (0.0:0.8)	1.5 * (1.0:2.0)	1.0 * (0.6:1.3)	2.6 * (2.2:3.0)
Meat	−0.5 (−1.2:0.1)	−0.5 (−1.3:0.3)	−2.1 * (−2.5:−1.7)	−1.9 * (−2.3:−1.4)	−1.2 * (−1.6:−0.9)	−1.5 * (−1.9:−1.0)
Dairy	−1.9 * (−2.5:−1.2)	−2.5 * (−3.4:−1.7)	−2.5 * (−3.0:−2.1)	−4.0 * (−4.5:−3.5)	−2.3 * (−2.7:−2.0)	−3.6 * (−4.0:−3.2)
Discretionary	4.7 * (3.9:5.5)	8.6 * (7.6:9.6)	4.8 * (4.3:5.4)	8.5 * (7.9:9.1)	5.5 * (5.0:5.9)	8.7 * (8.1:9.2)
Fluid	0.8 * (0.4:1.3)	4.1 * (3.5:4.7)	0.9 * (0.6:1.1)	4.0 * (3.7:4.2)	1.4 * (1.2:1.7)	4.1 * (3.8:4.3)
Variety	1.4 * (1.0:1.7)	5.0 * (4.5:5.4)	1.2 * (1.0:1.4)	3.9 * (3.7:4.2)	1.3 * (1.1:1.5)	4.2 * (4.0:4.4)
Healthy fats	2.4 * (1.7:3.2)	7.2 * (6.3:8.2)	3.6 * (3.1:4.1)	7.7 * (7.2:8.3)	3.6 * (3.2:4)	7.7 * (7.2:8.1)
Healthy Diet Score	1.9 * (1.6:2.2)	5.4 * (4.9:5.8)	1.8 * (1.5:2.0)	4.5 * (4.3:4.7)	2.2 * (2:2.4)	4.8 * (4.6:5.0)

^1^ Difference is normal weight minus overweight or obese. So positive score means normal weight has a higher score and therefore greater compliance with Dietary Guidelines. * *p* < 0.001.
